# Variability and harshness shape flexible strategy-use in support of the constrained flexibility framework

**DOI:** 10.1038/s41598-024-57800-w

**Published:** 2024-03-27

**Authors:** Sarah Pope-Caldwell, Dominik Deffner, Luke Maurits, Terrence Neumann, Daniel Haun

**Affiliations:** 1 Department of Comparative Cultural Psychology, Max Planck Institute for Evolutionary Anthropology, Leipzig, Germany; 2 Center for Adaptive Rationality, Max Planck Institute for Human Development, Berlin, Germany; 3 Science of Intelligence Excellence Cluster, Technical University Berlin, Berlin, Germany; 4 McCombs School of Business, University of Texas, Austin, TX USA; 5 Leipzig Research Center for Early Child Development, Leipzig, Germany

**Keywords:** Cognitive flexibility, Constrained flexibility framework, Decision-making, Adaptive cognition, Human behaviour, Cultural evolution

## Abstract

Human cognition is incredibly flexible, allowing us to thrive within diverse environments. However, humans also tend to stick to familiar strategies, even when there are better solutions available. How do we exhibit flexibility in some contexts, yet inflexibility in others? The constrained flexibility framework (CFF) proposes that cognitive flexibility is shaped by variability, predictability, and harshness within decision-making environments. The CFF asserts that high elective switching (switching away from a working strategy) is maladaptive in stable or predictably variable environments, but adaptive in unpredictable environments, so long as harshness is low. Here we provide evidence for the CFF using a decision-making task completed across two studies with a total of 299 English-speaking adults. In line with the CFF, we found that elective switching was suppressed by harshness, using both within- and between-subjects harshness manipulations. Our results highlight the need to study how cognitive flexibility adapts to diverse contexts.

## Introduction

Supported by an array of tools and behaviors, humans occupy and have modified over 80% of earth’s landmass ranging from arctic tundra to tropical forests^1,2^ Our ability to adapt to such diverse environments hinges upon two, often opposing, psychological skill sets: (i) the maintenance and faithful transmission of working strategies, and (ii) innovation and flexible updating or switching to better strategies when they are available^3,4^. The cognitive mechanism underlying the capacity to adaptively select between known solutions and innovated or acquired novel solutions in a contextually appropriate manner is termed cognitive flexibility^5–7^. As a long-lived, historically nomadic, social species, humans evolved to navigate the many changing ecological and social conditions they experience within a lifetime. Yet, the specific contexts which compel or constrain flexible and productive strategy-switching remain largely unclear.

When a previously successful strategy stops working (e.g., a favorite watering hole has dried up), it is always beneficial to change tact. Strategy-switching that occurs in response to failure, or anticipated failure— herein referred to as responsive switching—is considered a core executive function and has been extensively studied in both children and adults^8–10^. Common responsive switching tasks make use of forced-switch contexts, where participants are required to change strategies on command [e.g., the Color-Shape task^11^, Dimensional Change Card Sort task^12^] or after a previously effective strategy stops working [e.g., Reversal Learning tasks^13^, Wisconsin Card Sorting Task^14,15^]. Flexibility, or rather inflexibility, is then measured as the latency to adopt the new working strategy. From as young as 3 years of age, children exhibit proficient responsive switching under certain conditions^16^, but this skill continues to develop, reaching adult-like performance by around fifteen years of age^17^. Responsive switching has been studied extensively; however, in real-life, strategies do not always just simply stop working. More often, a current strategy exists alongside many possible alternatives and our ability to select between them is critical to adaptive decision-making.

Termed elective flexibility, deciding when, and when not, to switch away from a working strategy in order to sample alternatives is complex. The decision to switch away from a working strategy—herein referred to as elective switching—has the potential to lead to the discovery or innovation of a more efficient alternative, or it could result in wasted efforts and missed opportunities. If all possible strategies are known and their outcomes readily discerned then optimal behavior is simply a matter of choosing the best one. Yet, we live and have evolved within variable, ambiguous environments. If one were to assess each available strategy, determining the time and effort required to learn and implement them as well as quantifying all of their outcomes, any benefit gleaned from adopting the best strategy could easily be outweighed by the effort spent finding it—at least in the short term. Thus, determining when to electively switch strategies can be quite cognitively demanding.

One way to decrease the cognitive load associated with decision-making under uncertainty, as is the case for elective flexibility, is by using heuristics. Heuristics are rules-of-thumb or biases that are based on specific information or cues, like relative price or brand familiarity, that reduce cognitive effort when making a decision. Behavior guided by heuristics should, on the whole, result in reasonably sufficient outcomes, so long as the context in which the rule was formed does not differ substantially from the one in which it is deployed^18,19^. However, these cognitive shortcuts come at a cost. Heuristics frequently result in predictable biases and judgment errors^20^.

Consistent with this, when faced with the complex decision to electively switch from a working strategy and adopt an alternative, we tend to miss the mark. Cognitive set bias, the tendency to persist with a familiar strategy despite the availability of a better alternative, is found in a range of cognitive domains including mathematics^21–24^, design and engineering^25,26^, strategic reasoning^27^, tool-use^28,29^, as well as insight^30,31^, lexical^24,32^, and sequential problem-solving^33–38^. For example, in the Learned Strategy-Direct Strategy task only ~ 1 in 10 Americans between the ages of 7–68 electively switched from a learned solution to a better alternative^34,36^. Even after watching a video demonstrating the better strategy, many American undergraduates did not relinquish their familiar strategy^37^. However, intriguingly Pope et al.^35^ found that semi nomadic Namibian Himba pastoralist adults were ~ 4 times more likely to find and use the more efficient strategy than American adults. Interestingly, children may show heightened elective switching under certain circumstances^15,34^, in line with a general tendency towards exploration during childhood^39,40^; however, this has also recently been shown to be culturally variable^41^. To understand why humans exhibit flexible behavior in some contexts, yet striking inflexibility in others, we must consider the heuristics underlying elective flexibility, and how these are calibrated to optimize outcomes in various decision-making environments.

**Figure 1:**
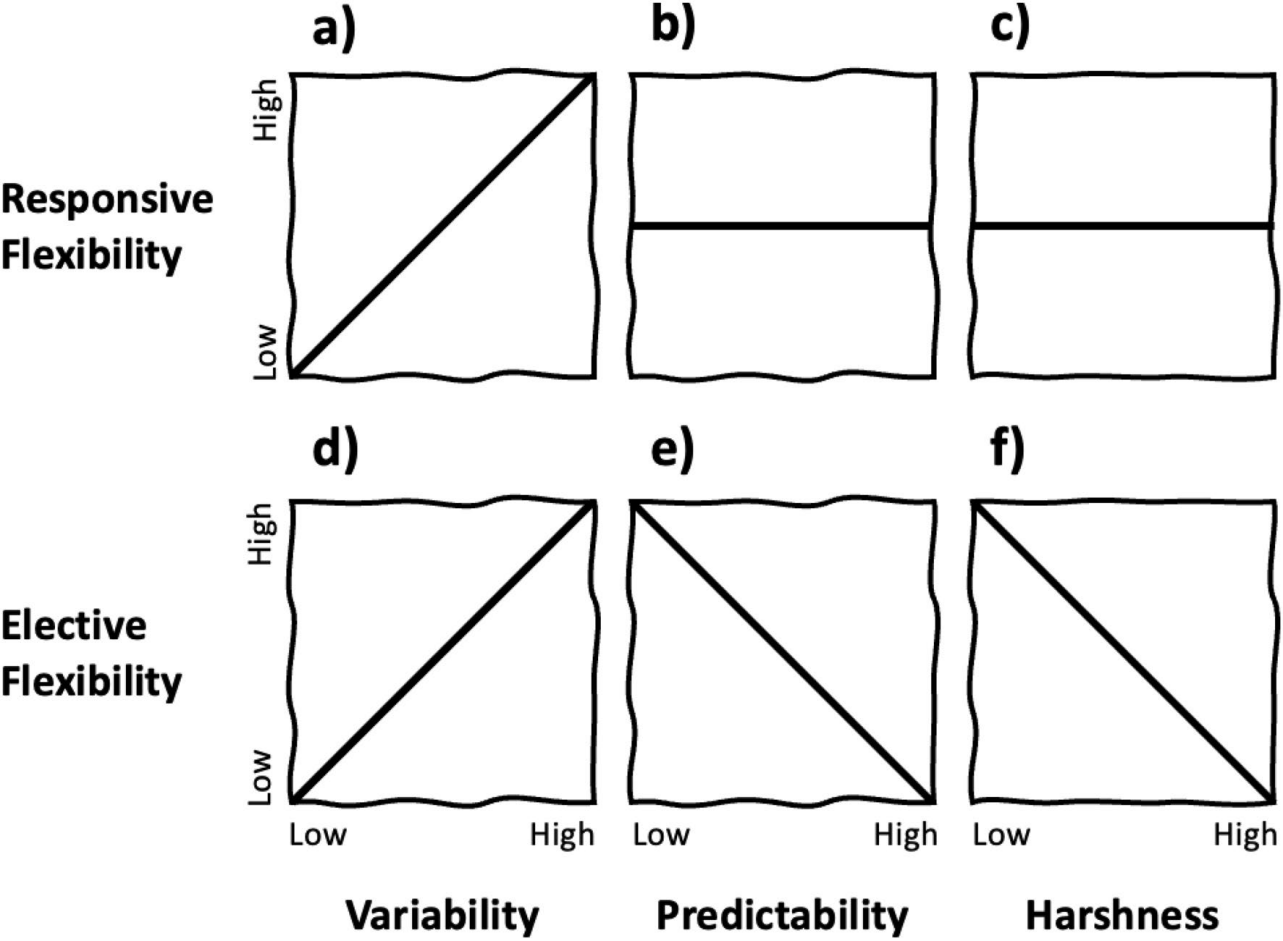
The impacts of variability, predictability, and harshness on responsive and elective switching, as described by the CFF. Responsive switching occurs when strategy efficacy is low, or failing. Elective switching is sampling alternatives despite strategy efficacy being high. a) As variability increases from stable to variable, responsive switching occurs more frequently, in response to strategy failures. However, responsive switches should occur whenever failure occurs or is imminent, regardless of whether b) the change in efficacy is predictable or c) the consequences of failure are high. d) Elective switching is also increasingly beneficial with increasing variability because the current strategy’s efficacy might be surpassed by an alternative. e) Yet, in predictably variable environments, elective switching is less valuable because after an initial sampling period, the best alternatives are already identified (although preemptive switching from effective strategies to soon-to-be effective alternatives is possible). f) Under conditions of high harshness, elective switching is suppressed to minimize the risk of failure.

To address this question, Pope-Caldwell^42^ proposed the constrained flexibility framework (CFF), a theoretical account of how elective flexibility is shaped by variability, predictability, and harshness. Variability is the extent to which the environment (i.e., problem-space) changes over time and space. Predictability is the temporal regularity of changes or the degree to which they are correlated. Harshness is exposure to factors that increase the severity of the consequences elicited by strategy failure^43,44^. The CFF asserts that high elective switching is mal-adaptive in stable or predictably variable environments, but may be very adaptive (i.e., result in finding better solutions and payoffs) in unpredictable environments, so long as harshness is low. When harshness is high, the costs of switching away from a working strategy are prohibitive (Fig. 1). To expand on this, consider that in a perfectly stable environment strategy outcomes do not change. Once an ideal strategy is adopted, there is no benefit to seeking or trying alternatives. However, in changing environments, an ideal strategy at one point might easily be usurped by an alternative in the next. When variation is predictable, changes in strategy efficacies can be detected or anticipated. Strategy switching in predictable environments should occur only in response to reliable indications that a current strategy has been, or is soon to be, eclipsed by an alternative (e.g., responsive switching). In contrast, unpredictably variable environments are ripe with opportunity for current strategies to be surpassed by alternatives. In this case, finding the best strategy at any point is a balancing act between maintaining an effective strategy while also monitoring alternatives. Here, adaptive behavior is supported by higher rates of elective switching—unless the consequences of failure are high. In harsh environments, any effective strategy is preferable to failure, regardless of variability and predictability.

Support for the CFF comes from diverse fields. For example, in decision-making and foraging tasks where participants must choose to either exploit a current resource or explore alternatives, more exploratory or information-seeking behavior is observed in variable compared to stable contexts^45–49^ and this effect is more pronounced in less predictable decision-environments^50–52^. Although the impact of harshness on elective flexibility is much less studied, a pronounced suppression of explorative behavior in response to risk is observed in both humans (e.g., loss-aversion bias^53^) and nonhuman animals^54^. For example, in their recent study, Shulz et al.^55^ found that in a maximization task participants made safer, less explorative choices under risky conditions. Additionally, reductions in exploration are observed in response to shorter time horizon^50,56,57^. Beilock & Decaro^58^ found that, when solving math problems, undergraduate participants who used complex solutions in low-pressure situations reverted to simpler strategies when pressure was high (see also:^59,60^). Despite substantial cumulative support for the CFF (for an expanded discussion:^42^), there has not been direct investigation of the role of harshness in mediating elective and responsive switching.

The current study aimed to test two core hypotheses stemming from the CFF:

1. Responsive switching occurs infrequently in stable environments and is not affected by harshness.
2. Elective switching occurs more frequently in variable compared to stable environments but is reduced by harshness in both.

**Figure 2:**
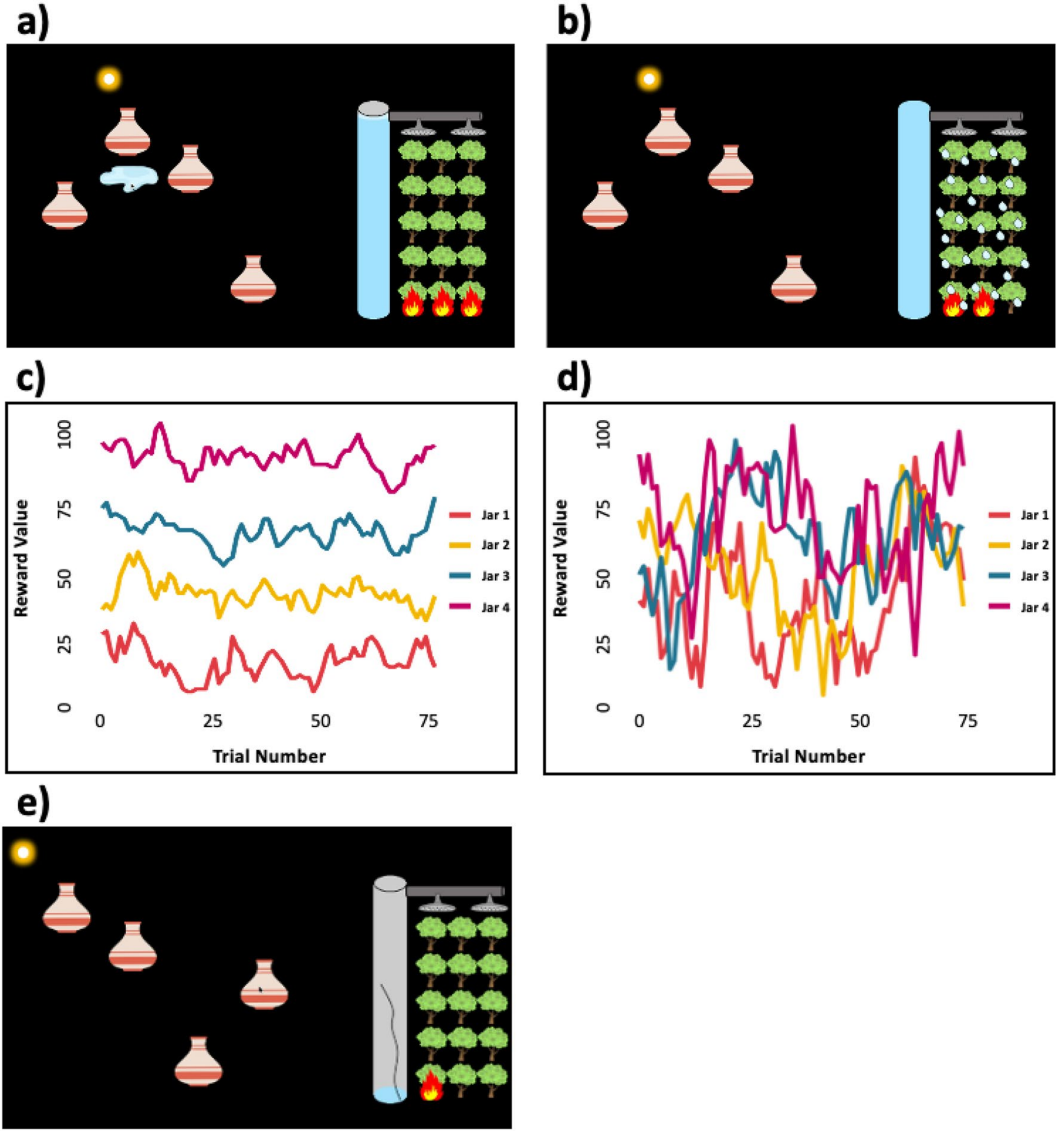
Task design for the Bandit Jars task. a) On each trial, participants selected any of the four jars and collected the water it yielded by selecting the puddle. Puddle-size corresponded to the amount of water that was added to the tube. b) Each time the tube was filled, a rain shower was produced, putting out one of the fires. Fires regenerated or spread after every three trials, with rain showers resetting the count. The amount of water each jar produced was predetermined by an underlying reward schedule which was either c) stable (i.e., jar yields were consistent relative to one another) or d) variable (jar yields fluctuated drastically) over the course of the 75-trial blocks. e) In the Harsh condition, the tube was cracked and leaked after every selection, making it more difficult to fill the tube.

In Study 1, using an online four-armed bandit decision-making task, we analyzed 100 English-speaking adult participants’ ability to adaptively select the highest-yielding water jars in order to put out a simulated forest fire (Fig. 2a,b) across four conditions: Stable Not Harsh, Stable Harsh, Variable Not Harsh, and Variable Harsh. In stable conditions, the reward schedules underlying each jar did not change substantially over time (Fig. 2c). In variable conditions, reward schedules changed dramatically over time (Fig. 2d). In the Harsh condition, the tube used to store the water leaked after each selection, increasing the value of higher jar-yields (Fig. 2e). In the Not Harsh condition, the tube did not leak. After completing the final trial block, participants were asked to self-report the level of stress they experienced during the Harsh and Not Harsh conditions.

In Study 2, we used the same procedure as Study 1 but with an increased sample size (199 English-speaking, middle-income adults) and created monetary performance incentives. Specifically, participants received an additional £0.03 each time they filled the tube.

### Study 1 Results

#### Study 1 General results

Participants chose the optimal jar, determined on a trial-by-trial basis based on the current reward values, more often than would be predicted by chance (chance proportion of optimal choices = 0.25) for all combinations of environment and condition (Stable-Not Harsh: 95% highest posterior density interval (HPDI) = [0.94, 0.96]; Stable-Harsh: 95% HPDI = [0.95, 0.96]; Variable-Not Harsh: 95% HPDI = [0.39, 0.43]; Variable-Harsh: 95% HPDI = [0.39, 0.43]). Additionally, participants made better choices over time (later trial numbers predicted higher average cumulative scores) for all combinations of environment and condition (Stable-Not Harsh: 95% HPDI = [14.54, 15.65]; Stable-Harsh: 95% HPDI = [13.85, 14.94]; Variable-Not Harsh: 95% HPDI = [0.18, 1.30]; Variable-Harsh: 95% HPDI = [3.37, 4.48]).

#### Study 1 Perceived harshness results

Out of 100 participants, 69% reported feeling more stressed in the Harsh compared to the Not Harsh condition, 30% reported no difference between the conditions, and 1% reported feeling more stressed in the Not Harsh compared to the Harsh condition (Supplementary Fig. 1). A Bayesian ordinal regression indicated that participants reported feeling 2.19 standard deviations more stressed in the Harsh compared to the Not Harsh condition (95% CI = [1.72, 2.71]).

#### Study 1 Switching results

We predicted that the likelihood of switching away from a given strategy (using a specific jar) would be impacted by (i) the quality of the previous reward obtained using that strategy (i.e., the quantity of water last yielded from that jar), (ii) the variability of the rewards generated by all possible strategies (i.e., how much the quantity of water gained from each jar selection changed over time), and the consequences of not generating an adequate reward on any given selection (i.e., how much water needed to be found to extinguish the fires—recall this is much higher in the harsh condition due to the leaking tube). Stemming from our first hypothesis, we expected the likelihood of switching following high-value rewards (i.e. elective switching) to be higher in Variable Not Harsh conditions than in Stable Not Harsh conditions, reflecting the increased need to explore when reward dynamics are in flux. However, in both Variable and Stable Harsh conditions we expected elective switching to be reduced, reflecting the heightened consequences of failure. From our second hypothesis, we expected that switching following low-value rewards (i.e., responsive switching) would be infrequent in Stable environments but not affected by harshness in either Stable or Variable environments.

**Figure 3:**
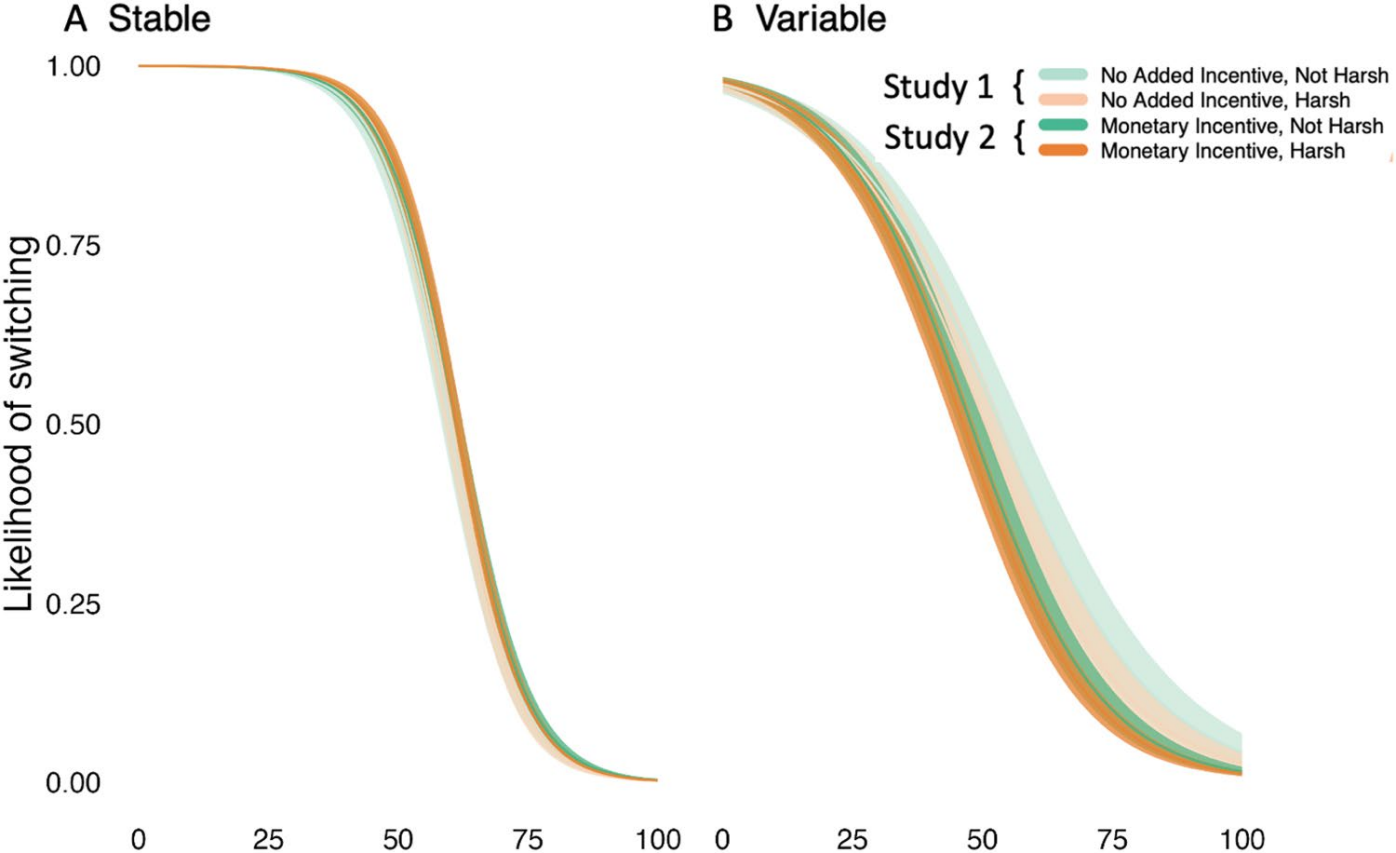
The likelihood of switching jars (y-axis) after finding reward values from 0–100 (x-axis) in Study 1: No Added Incentive—Not Harsh, No Added Incentive – Harsh and Study 2: Monetary Incentive—Not Harsh and Monetary Incentive—Harsh conditions in A) Stable and B) Variable environments. Recall that responsive switching is switching after finding low value rewards, while elective switching is switching that occurs after finding high value rewards.

We ran a series of Bayesian Binary Logistic Regressions which indicated that participants’ switching behavior was impacted by a three-way interaction between variability, harshness, and the reward value (Fig. 3; Supplementary Table 1). We broadly identified elective and responsive switching as switches that occurred immediately after finding high (55–100) and low (0–45) value rewards, respectively. Posterior distributions (Methods) analyzed for differences in switching probabilities indicated that in stable conditions, responsive switching was slightly reduced in Not harsh compared to Harsh conditions [95% HPDI = (−0.05, 0.00)]; however, there was no clear effect of harshness on elective switching [95% HPDI = (−0.08, 0.03)]. Thus, contrary to our prediction that responsive switching would be unaffected by harshness, participants may have been slightly less tolerant of low-value rewards in the Stable Harsh compared to Stable Not Harsh condition (see discussion). By contrast, for variable conditions, there was no clear impact of harshness on responsive switching [95% HPDI = (−0.03, 0.07)]; however, the likelihood of elective switching seems to have been slightly higher in Not Harsh compared to Harsh conditions [95% HPDI = (−0.02, 0.11)]. This would suggest that, in line with the CFF, participants switched away from higher value rewards less in the Harsh compared to the Not Harsh condition. Supplementary analyses confirmed that results were not impacted by participants’ age and sex, nor the specific set of reward schedules they encountered (Supplementary Table 2).

We also reran these analyses using only those participants who reported that the Harsh condition was more stressful than the Not Harsh Condition. These supplementary analyses did not differ meaningfully from the original findings, other than the effect of harshness in suppressing elective switching was more pronounced [95% HPDI = (0.00, 0.15)]; Supplementary Table 3). Finally, these results were supported by supplementary frequentist analyses using the *lme4* package version 1.1.35.1^61^ in R^62^ (see the analyses script available in our Github repository).

#### Study 1 Reinforcement learning model results

One limitation of simply measuring switching behavior is that switching can occur both away from a preferred strategy, or back to it. Indeed, returning to a favored strategy after an explorative choice is not itself indicative of elective flexibility, even though the previous choice might have been^46^. Thus, in addition to our originally planned switching analyses, we decided to gain a better understanding of how participants’ choices reflected their accumulated knowledge (based on their previous selections) about jar values in each condition and environment^63^. To accomplish this, we ran a Bayesian multi-level reinforcement learning model as implemented by Deffner et al.^64^ and also used in^65,66^. For each participant, in each trial block–Stable Not Harsh, Stable Harsh, Variable Not Harsh, and Variable Harsh–we calculated (i) a *learning rate* parameter based on how quickly participants learned about the jars' reward values, and (ii) an *elective exploration* parameter based on their willingness to explore jars other than the one with the estimated best-payoff at each time point. We inferred differences in parameters across conditions by computing respective contrasts from their posterior estimates (Methods).

Based on our hypotheses, we expected that elective exploration would be enhanced in Variable Not Harsh conditions compared to Stable Not Harsh conditions, again, reflecting the increased value of exploration when reward values are changing. However, in both Variable and Stable Harsh conditions we expected elective exploration to be suppressed by the heightened consequences of failure. We did not have any de facto predictions regarding the learning parameter. It seemed reasonable to expect that faster learning would be most beneficial in Variable compared to Stable environments as well as in Harsh compared to Not Harsh conditions.

**Figure 4:**
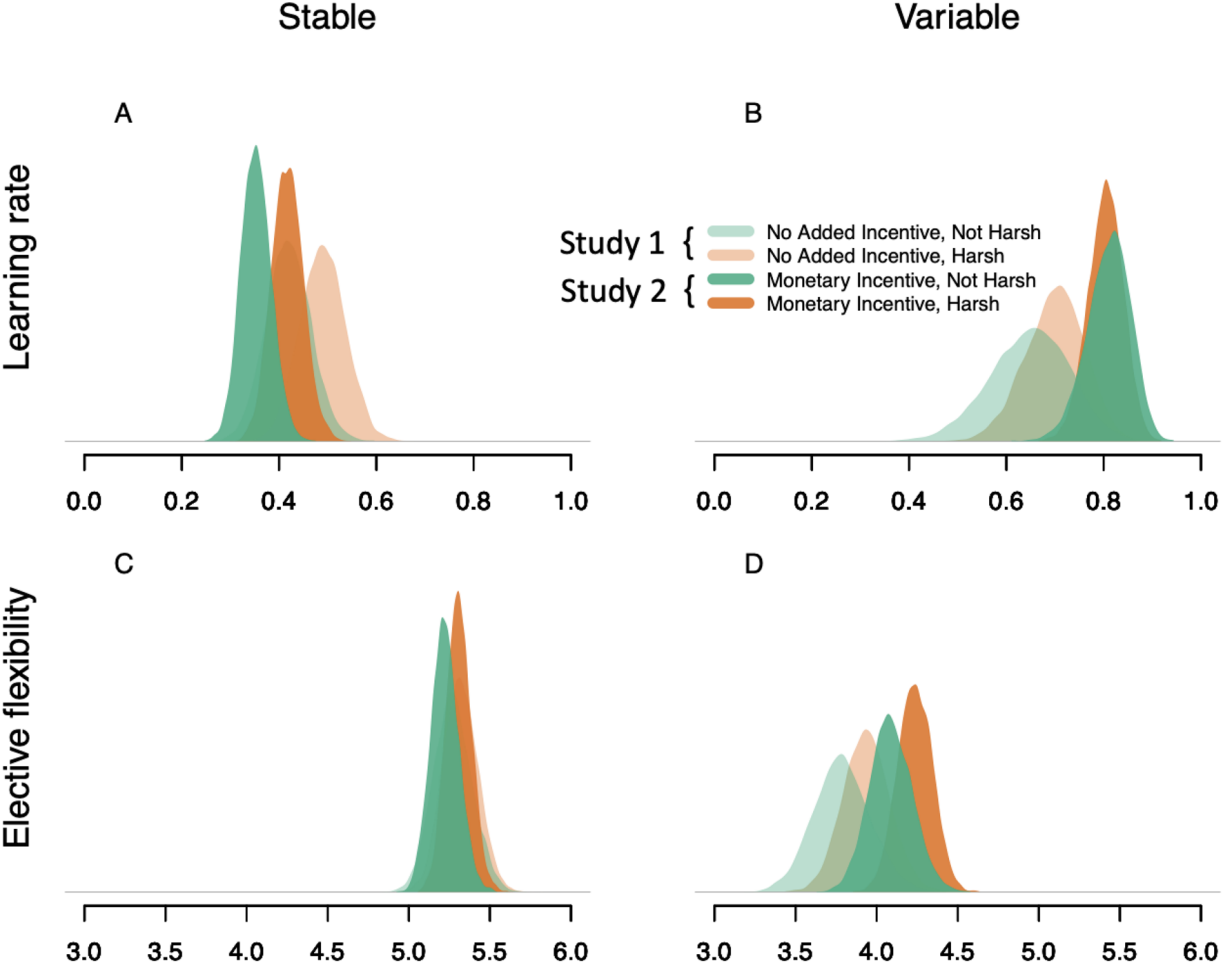
Studies 1 and 2 Reinforcement Learning Model results. Posterior probability distributions in Stable and Variable reward environments for participants’ learning rate (A-B) and elective exploration parameters (C-D) in Study 1: No Added Incentive—Not Harsh, No Added Incentive—Harsh and Study 2: Monetary Incentive—Not Harsh and Monetary Incentive—Harsh conditions.

The reinforcement learning model results (Fig. 4) mirrored those of the switching analyses, indicating an interaction between variability and harshness on participants’ learning and elective exploration. Specifically, participants tended to learn faster in high harshness conditions, with a slightly more pronounced difference in stable environments [Posterior Harsh-Not Harsh contrast 95% HPDI = (−0.04, 0.18)] compared to variable environments [95% HPDI = (−0.09, 0.19)]. We also found some evidence suggesting that elective exploration was reduced in the high harshness condition in variable environments [95% HPDI = (−0.15, 0.45)] but not in stable environments [95% HPDI = −0.21, 0.32)]. Note that lower values of the model parameter indicate higher elective exploration. Thus, the reinforcement learning results mirror those of the switching analyses. Repeating this analysis only for participants who reported experiencing the Harsh condition as more stressful than the Not Harsh condition produced overall similar inferences, but revealed more pronounced differences in learning [95% HPDI Stable = (−0.04, 0.22); Variable: = (−0.05, 0.26)] and elective exploration [95% HPDI Stable = (−0.21, 0.40); Variable: = (−0.08, 0.59)] between harshness conditions compared to the full sample (Supplementary Fig. 2).

### Study 2

In response to reviewer suggestions, we ran a follow-up study to further increase harshness by adding real-world stakes. To this end, Study 2 participants received £0.03 each time they filled the tube. Additionally, we increased the goal sample size to 200; however, this was reduced to 199 after excluding participants who took longer than 45-min to complete the task (N = 2; see methods for details).

#### Study 2 General results

Like Study 1, participants chose the optimal jar more often than would be predicted by chance for all combinations of environment and condition (Stable-Not Harsh: 95% HPDI = [0.94, 0.96]; Stable-Harsh: 95% HPDI = [0.94, 0.96]; Variable-Not Harsh: 95% HPDI = [0.41, 0.44]; Variable-Harsh: 95% HPDI = [0.42, 0.45]). Additionally, participants made better choices over time for all combinations of environment and condition (Stable-Not Harsh: 95% HPDI = [13.65, 14.44]; Stable-Harsh: 95% HPDI = [14.47, 15.26]; Variable-Not Harsh: 95% HPDI = [1.86, 2.65]; Variable-Harsh: 95% HPDI = [1.23, 2.02]).

#### Study 2 Perceived harshness results

Out of 199 participants, 69% reported feeling more stressed in the Harsh compared to the Not Harsh condition, 27% of participants reported no difference between the conditions, and 4% of participants reported feeling more stressed in the Not Harsh compared to the Harsh condition (Supplementary Fig. 1). A Bayesian ordinal regression indicated that participants reported feeling 1.76 standard deviations more stressed in the Harsh compared to the Not Harsh condition (95% CI = [1.48, 2.05]).

#### Study 2 Switching results

##### Harsh vs not harsh

In contrast to Study 1, responsive switching did not differ between Not harsh and Harsh conditions in the Stable reward environment [95% HPDI = (−0.01, 0.01)]; in other words, once monetary incentives were introduced, participants were no longer tolerant of lower value rewards in the Stable condition. However, all other contrasts between Harsh and Not Harsh conditions were in line with Study 1. In the Stable reward environment, elective switching did not differ between Stable Not Harsh and Stable Harsh conditions [95% HPDI = (−0.02, 0.06)]. Additionally, in the Variable reward environment there was no clear impact of harshness on responsive switching [95% HPDI = (−0.02 0.06)]; but elective switching was again slightly higher in Not Harsh compared to Harsh conditions [95% HPDI = (−0.00, 0.06)]. Thus, for both Studies 1 and 2, the within-study harshness manipulation (e.g., when the tube cracked) slightly reduced elective switching in the Variable reward environment (Fig. 3; Supplementary Table 5).

##### Monetary incentive vs no monetary incentive

To assess how adding monetary incentives and therefore increasing the overall harshness (i.e., the consequences of failure) impacted elective switching, we compared participants’ jar-switching behavior between Study 1 and Study 2. We found that participants’ switching behavior was best predicted by a four-way interaction between incentive, variability, harshness, and the reward value (Supplementary Table 6).

In Stable reward environments, responsive switching did not differ between participants with No Monetary Incentive (Study 1) and those with a Monetary Incentive (Study 2), for either Harsh [95% HPDI = (−0.01, 0.01)] or Not Harsh [95% HPDI = (−0.02, 0.01)] conditions. There was also no difference in elective switching in Stable reward environments for both Harsh [95% HPDI = (−0.09, 0.03)] and Not Harsh [95% HPDI = (−0.10, 0.03)] conditions. In Variable reward environments, responsive switching was similarly unaffected by the monetary incentive in both Harsh [95% HPDI = (−0.03, 0.11)] and Not Harsh [95% HPDI = (−0.03, 0.12)] conditions. However, elective switching in Variable reward environments was significantly higher for participants receiving No Monetary Incentives, in both Harsh [95% HPDI = (0.00, 0.14)] and Not Harsh conditions [95% HPDI = (0.01, 0.19)]. Thus, in line with the CFF, elective switching was suppressed when money was on the line, and therefore the overall consequences of failure (between-study harshness) was higher (Fig. 3).

Supplementary analyses confirmed that results were not impacted by participants’ age and sex, nor the specific set of reward schedules they encountered (Supplementary Table 7). We also reran these analyses using only those participants who reported that the Harsh condition was more stressful than the Not Harsh Condition. These supplementary analyses did not differ meaningfully from the original findings, other than the effect of incentive in suppressing elective switching in Variable reward environments was slightly less pronounced in Harsh conditions [95% HPDI = (−0.02, 0.10); Supplementary Table 8]. Finally, these results were supported by supplementary frequentist analyses using the *lme4* package version 1.1.35.1^61^ in R^62^ (see the analyses script available in our Github repository).

#### Study 2 Reinforcement learning model results

The reinforcement learning model results for Study 2 (Fig. 4) suggest that monetary incentives led to slightly slower learning rates in Stable reward environments for both Harsh [95% HPDI = (−0.17, 0.05)] and Not Harsh [95% HPDI = (−0.17, 0.04)] conditions; yet, faster learning rates in Variable reward environments, for both Harsh [95% HPDI = (−0.03, 0.24)] and Not Harsh [95% HPDI = (−0.01, 0.33)] conditions. Interestingly, and in line with the CFF, participants’ elective exploration was unaffected by the monetary incentives in Stable reward environments for both Harsh [95% HPDI = (−0.26, 0.24)] and Not Harsh [95% HPDI = (−0.34, 0.23)] conditions but in Variable reward environments, the monetary incentive seems to have suppressed elective exploration in both Harsh [95% HPDI = (−0.05, 0.63)] and to some extent in Not Harsh [95% HPDI = (−0.14, 0.69)] conditions. Repeating this analysis for only those participants who reported experiencing the Harsh condition as more stressful than the Not Harsh condition produced no substantive changes (Supplementary Fig. 2).

## Discussion

In Study 1, we measured the impact of a within-study harshness manipulation (Harsh vs Not Harsh) on participants’ propensity to exploit a current strategy and / or explore alternatives in a four-armed bandit decision-making task in both Stable and Variable conditions. In Study 2, we further increased the overall consequences of failure by implementing a monetary performance incentive, effectively constituting a between-study (Study 1 vs. Study 2) harshness manipulation. The CFF posits that elective switching and sampling of alternatives should occur most frequently in variable but not harsh contexts. In line with the CFF, both Studies 1 and 2 found that participants’ (i) elective switching, evidenced by their propensity to change jars immediately after finding a high-value reward and (ii) elective exploration, their willingness to explore sub-optimal jars based on their own prior experience, was slightly suppressed by the within-study harshness manipulation in Variable but not Stable environments, where it was already quite low. Additionally, and again in line with the CFF, Study 2 found that increasing the overall harshness, by instating monetary incentives, led to less elective switching and lower RLM-derived elective exploration parameters compared to Study 1, in Variable but not Stable conditions. Furthermore, this effect seems to have been additive, as the differences were most prominent for the within-study Harsh condition. In summary, and in support of the CFF, across both Studies 1 and 2 participants favored reliable strategies when harshness was high and when the likelihood of finding a better strategy was low (i.e., stable environment).

We originally predicted that responsive switching, switching away from a failing strategy, would be immune to the harshness manipulation and, at first glance, this runs contrary to our Study 1 finding that, in Stable conditions, switching after finding a low-value reward was slightly reduced in the Harsh compared to Not Harsh condition. However, recall that we coarsely defined responsive switching as switches occurring after any reward value 45 units or less. It is likely that participants did not consider rewards at the top end of this range as indicative of strategy failure, and may have been more tolerant of somewhat lower value rewards in the Stable Not Harsh condition, perhaps because better alternatives were easily identifiable when needed (see Supplementary Tables 1d and 5d for additional responsive switching analyses with reward values 25 units or less). Furthermore, with the addition of monetary incentives in Study 2, elective switching in the Stable condition was no longer higher for the within-study Not Harsh condition, suggesting that participants were no longer tolerant of lower value rewards when money was on the line.

The effect of harshness and monetary incentives on learning rates is somewhat paradoxical. In Study 1, learning rates were quite low in the Not Harsh compared to the Harsh Stable condition, suggesting that, without harshness, participants took longer to accumulate information about jar values. Although a similar trend in learning rate can be seen in the Variable conditions, the effect was less pronounced—perhaps a reflection of the greater need to continuously update, regardless of harshness, in the Variable environments^67^. We observed a similar increase in learning rates when the monetary incentive was introduced in Study 2, but only for the Variable reward environment; in the Stable condition, learning rates were slower compared to Study 1. Thus, paradoxically, for Stable conditions the within-study harshness increased learning rates but the monetary incentive “harshness” actually slowed learning rates. It is unclear why we observed this pattern of results; future studies would benefit from implementing reward structures better representing a range from stable to variable.

Here, we found that elective switching and sampling were substantially lower for participants exposed to the increased consequences of failure (i.e. harshness) associated with monetary incentives but only slightly reduced by the within-study harshness manipulation. However, especially considering that the experiment was formatted as an online game, our within-study harshness manipulation may not have been perceived as truly harsh—in the sense that the real-life consequences of failure were not high. How strongly harshness is expected to influence behavior of course depends on how harsh conditions are actually perceived by participants. In fact, only 39% of participants in Study 1 and 37% in Study 2 reported being more than “slightly stressed” during the within-study Harsh condition. Supporting the importance of perceived harshness, when analyzing the subset of participants (69%) who reported that the Harsh condition was more stressful than the Not Harsh condition in Study 1, the impact of harshness on elective flexibility was enhanced (evidenced by both switching and RLM supplementary analyses). We did not find a similar strengthening of the effect of the within-study harshness manipulation for Study 2, although it is very possible that, for Study 2 participants, the perceived harshness imposed by the monetary incentive overshadowed that of the within-study harshness manipulation. Furthermore, although the differences in elective switching and sampling between within-study Harsh and Not Harsh conditions were slight, we argue that they are still to be considered substantive given that, unbeknownst to participants, the underlying reward schedules in Stable Harsh and Stable Not Harsh conditions were identical, as were Variable Harsh and Variable Not Harsh—meaning that all differences in behavior within these trial-block sets are directly attributable to the harshness manipulation.

Another limitation, inherent to online testing in general, is that it was not possible to confidently exclude participants who were distracted, unmotivated, or who might not have clearly understood the task, despite completing the instructions module. Indeed, two participants from both Study 1 (2%) and Study 2 (1%) switched jars every single selection, usually adhering to a pattern (e.g., Jars 3–2-1–4-3–2–1–4…), but as this only occurred in the variable condition it is unlikely that it can be attributed to misunderstanding the task goals. Rather, it may be indicative of these participants implementing meta-strategies to reduce cognitive load. In fact, Pope-Caldwell et al. (In Prep) recently found that children used these so-called meta-strategies to a much greater extent. Importantly, the current analyses would consider these patterned responses to be indicative of high elective switching and exploration, when in fact they may represent a more inflexible adherence to fixed meta-strategies. Future investigations will benefit from more direct investigation of meta-strategy use as well as in-person testing.

Finally, in the current study we chose not to manipulate predictability. This was primarily due to logistical constraints in manipulating both predictability and harshness within the same task design (Methods). However, recent work using contextual multi-armed bandit tasks^48,49^ may prove a fruitful avenue for this line of research.

### Further considerations

The contexts giving rise to flexible behavior are of substantial adaptive importance. Theories surrounding human cognitive evolution posit a range of conditions which may have contributed to the emergence of bigger brains, better toolkits, flexible social skills, etc., including variable environments (*cognitive buffer hypothesis*:^68,69^; *brain size–environmental hypothesis:*^70^; see also:^71–74^, novel or otherwise unpredictable environments (*behavioral drive hypothesis:*^75^; and *adaptive flexibility hypothesis:*^76^), and harsh environments (*habitat theory:*^77,78^; *savannah hypothesis:*^79,80^; and *niche construction hypothesis:*^81^; see also^82^). Although the CFF is not the first to propose roles for variability, predictability, and harshness in shaping adaptive cognition, it is unique in its consideration of their combined effects and how they might explain variation in individuals’ decision-making across contexts. The CFF accounts for both flexibility that occurs in response to necessity (*necessity is the mother of invention hypothesis:*^83,84^)—what would be classified as responsive switching within the CFF—as well as the seemingly opposing idea that innovations occur in times of low stress (*spare time hypothesis:*^80^)—the increase in elective switching which occurs in the absence of harshness. Findings from the current study demonstrate the essential need to consider how variability and harshness work in concert to promote and suppress flexible behavior.

The CFF posits that risk is reduced by limiting elective switching under conditions where the consequences of failure are high; however, this is not the whole story. Humans have evolved another–quite effective–means of mitigating the costs of elective switching. The ability to socially acquire information and skills is considered a key force in shaping the evolution of human life history, which seems geared towards benefitting from cultural traditions^85,86^. This is not surprising, as copying successful behaviors from other individuals can all but eliminate the risks associated with exploring an alternative strategy. The CFF posits that harshness is a strong suppressant of elective switching; however, this argument hinges on the notion that if the consequences of failure are high, then any risks of failure should be avoided. Acquiring information about alternative strategies through social learning allows one to weigh the apparent costs and benefits without personally risking failure. It is not surprising that humans often capitalize on socially-acquired strategies, especially when they are likely to be reliably useful^15,87,88^. Yet, leaning too heavily on social learning can also lead to the propagation of inefficient strategies^89,90^ (see also: overimitation^91,92^) and under-exploration of alternatives^41,93,94^. Considered alongside the CFF, we might expect that under conditions of harshness, elective switching would be rescued when social information about alternatives is available. Future research should investigate social information-use under dynamic, harsh conditions.

## Concluding remarks

In real decision-making environments, variability, predictability and harshness exist along multiple, concomitant spatiotemporal scales and exposure to their influence is mediated by our own behaviors and socio-cultural environments. Understanding flexible strategy-use under controlled dynamic conditions is necessary to parcel out the complex processes underlying real-life adaptive cognition. The current results provide direct support for the CFF, by showing that harshness suppresses elective switching and exploration in variable but not stable environments. A larger takeaway from these findings is that when stakes are high, decision-makers are likely primed for inflexibility. Thus, cultivating flexibility hinges on both identifying the possibility of better alternatives and consequences that are low-enough to try them.

## Methods

### General

This study was pre-registered prior to data collection^95^(see also^63,96^). Following an initial round of data collection, it was reported that a viral TikTok post^97^ one week prior had prompted a surge of young female Prolific participants, and this was reflected in our initial sample: 86.7% of respondents were female with a mean age of 22.6 (SD = 5.8). Due to the evident skew in demographics, and the TikTok video’s framing of Prolific participation as an easy “side hustle,” we decided to rerun the study to ensure data quality was not affected by the sample (see addendum to the preregistration:^94^). However, round one data are available here and analyses are included in Supplementary Table 4.

### Participants

Participants were recruited via Prolific, an online data collection resource with over 130,000 participants from around the world (www.prolific.co). The sample was limited to self-identified English-fluent participants, as instructions were given in English. For their data to be included, participants needed to pass the instructions portion of the experiment and complete all trials. All experiments were approved by the ethics committee at the Leipzig Research Center for Early Child Development and all experiments were performed in accordance with the relevant guidelines and regulations. Informed consent was obtained from all participants prior to data collection.

Study 1. Our goal sample size (100) was determined using simulated datasets of varying sample sizes, generated by sampling model parameters from our priors. We first confirmed the predicted best-fit model had the lowest WAIC value in over 95% of simulations and then analyzed for sign errors between the posterior mean estimates for each parameter and the known values used for the simulation. For 100 participants, there were no sign errors (i.e. every parameter’s posterior mean had the correct sign) in 80% of these simulations, so that we expected our fitted model to make correct qualitative claims about the directions in which different conditions shift participant behavior. All participants who attempted the task received £2.34 compensation for their efforts (the equivalent of £10.00 per hour based on the median time to completion: 13.91 min). Our final sample consisted of 100 (50 female) adults from Australia (N = 1), Canada (N = 21), Ireland (N = 2), Italy (N = 1), Poland (N = 1), South Africa (N = 3), Spain (N = 1), the United Kingdom (N = 54), and the United States (N = 16). The mean age was 28.3 years (Min = 18, Max = 59, SD = 9.7).

Study 2. Given the small effect size of the within-study Harshness manipulation in Study 1, we increased our goal sample size to 200; however, after excluding participants who took longer than 45-min to complete the task (N = 2; task durations = 6.1 and 20.9 h) the final sample was 199 participants. Although this exclusion criterion was not preregistered, we felt it would be imprudent to analyze these data alongside the rest. That said, their inclusion did not substantively influence our results. All participants who attempted the task received a base payment of £1.50 compensation for their efforts. Additionally, bonus payments were distributed based on the number of times participants filled the tube (Mean = £1.55, Min = £1.11, Max = £1.77). Thus, average total compensation was the equivalent of £11.08 per hour based on the median time to completion: 15.47 min). Our final sample consisted of 199 (99 female) adults from Australia (N = 7), Canada (N = 9), France (N = 1), Ireland (N = 6), Italy (N = 1), Poland (N = 2), Portugal (N = 1), South Africa (N = 25), Spain (N = 2), the United Kingdom (N = 140), and the United States (N = 5). The mean age was 38.7 years (Min = 19, Max = 79, SD = 13.5).

### Bandit jars task

In a four-armed bandit decision-making task, the Bandit Jars Task, Participants completed 300 trials across four blocks: Stable Not Harsh, Stable Harsh, Variable Not Harsh, and Variable Harsh. Condition order was pseudo-randomized such that either Stable or Variable environments appeared first, and within these sets either Harsh or Not Harsh conditions came first. In each 75-trial block, participants were presented with four jars, located randomly, but not overlapping, on the screen (see Fig. 2a). On the right of the screen a vertical tube and a patch of 15 trees was depicted. After every three trials, a fire engulfed one of the trees (accompanied by an “igniting” sound). Participants were instructed that the goal of the game was to “try to save trees from the fire” by collecting as much water as they could. Each trial started with participants selecting a jar, which was then overturned, releasing a puddle of water. The size of the puddle corresponded to an underlying reward schedule ranging from 1–100. Participants then clicked the puddle to collect it—the water level in the tube raised by the corresponding amount, and the trial was finished. When the tube reached 300 reward units, it appeared full and a brief rain storm was initiated (raindrops fell over the trees accompanied by a rain sound), reducing the number of active fires by one (Fig. 2b). Rains also reset the countdown to the next fire. The trial count (e.g., time horizon) was depicted by a sun moving across the screen (from left to right) incrementally after each trial. Prior to starting the game, participants completed interactive instructions where they demonstrated proficiency in (i) selecting the largest puddle, (ii) putting out fires, and (iii) estimating remaining time based on the sun’s location. Participants were allowed to repeat the instructions phase up to three times, after which the game automatically ended.

### Stable vs variable environment

We decided not to manipulate predictability in this study, as it would be better investigated using a different task design, one with longer trial-blocks for participants to learn predictive associations or more explicit cues regarding state shifts. Instead, we maximized the predicted impact of harshness by using a highly unpredictable reward structure to characterize the variable environment (see Fig. 2). For illustrative purposes, throughout the manuscript we use the labels Jar 1, Jar 2, Jar 3, and Jar 4 to represent the jars with the lowest to highest total (cumulative) reward schedule value. For the stable condition, this means that Jar 4 is the best option at all time points (Fig. 2c). For the variable condition, any jar could be the best jar at a given time point; however, Jar 4 had the highest cumulative reward value (Fig. 2d).

Both stable and variable reward schedules were generated using simulated autoregressive integrated moving average (ARIMA) models with the arima.sim function from the *stats* R core package (R Core Team, 2022; reward schedule code is available here). Acceptable variance ranges were set to 20–40 and 200–400 for stable and variable reward schedules, respectively. The acceptable range for the difference between jars’ cumulative reward value was set to 500–3000 units. To ensure that overall reward environments did not differ substantially between conditions, we set the acceptable range for total value of all four reward schedules to 14,500–15,000 units. Five sets of stable and variable reward schedules were generated, presentation of which was counterbalanced across participants. For each participant, and unbeknownst to them, the reward schedules underlying both Stable conditions (Harsh and Not Harsh) and both Variable conditions (Harsh and Not Harsh) were identical.

Prior to each round, participants were shown either the stable or variable jar type and told “Now let's play with these jars. These jars are special because the amount of water in each jar only changes a LITTLE over time. Let's practice! Select jars to see how the amount of water in each jar changes a LITTLE over time.” for the stable condition and “Now let's play with these jars. These jars are special because the amount of water in each jar changes a LOT over time. Let's practice! Select jars to see how the amount of water in each jar changes a LOT over time.” for the variable condition. This was followed by a 15-trial practice round in which participants could select between four jars equally spaced across the horizontal midline of the screen. The reward schedules underlying the practice jars were generated using the same parameters as the experimental trials’ and were the same for all participants.

### Not harsh vs harsh condition

In the Harsh condition, the tube which needed to be filled in order to put out fires cracked at the beginning of the round, resulting in it leaking 35 reward units per jar selection. The tube crack was accompanied by a glass breaking sound; Fig. 2e). Participants were told “Oh no! The tube cracked! Now each time you choose a jar, a little water will leak out. Like this:” followed by a brief demonstration of the tube leaking (a drop of water fell from the bottom of the tube and the fill line dropped). When a Not Harsh trial block followed a Harsh trial block, the participant was told “Oh good! The tube is fixed! Now each time you choose a jar, no water will leak out. Like this:” followed by a brief demonstration of the tube not leaking.

### Perceived harshness assessment

After completing the Bandit Jars Task, participants were asked to self-report the level of stress they experienced during the Harsh and Not Harsh conditions. First, they were presented with the image of the cracked tube and asked, “After the tube CRACKED, and water began to leak out, did you feel stressed?" Allowable responses were: 1—not at all stressed, 2—slightly stressed, 3—somewhat stressed, 4—moderately stressed, 5—extremely stressed. Next, they were presented with the image of the undamaged tube and asked, “After the tube was FIXED, and water did not leak out, did you feel stressed?" Allowable responses were the same.

### Study 1 Switching analyses

We ran a series of Bayesian Binary Logistic Regressions using the *brms*^98,99^ package in R^62^ to predict participants’ likelihood of switching jars: a binary measure of whether or not participants' chose the same jar (0) or a different jar (1) in the next trial. Model 1.0 served as a baseline, including only per-participant random intercepts. Model 1.1 also included a main effect of the value of the most recent reward found (reward value; e.g., the size of the puddle collected in that trial). Model 1.2 allowed the intercept and reward value slope to depend on the variability of the environment [Variable (1) vs Stable (0)] and the harshness condition [Harsh (tube cracked; 1) versus Not Harsh (0)], with maximal random effects structure but no interaction between variability and harshness. Model 1.3 allowed the intercept and reward value slope to depend also upon the interaction between variability and harshness. Based on our hypotheses and predictions, we expected Model 1.3 to best fit the data. To determine the best fit model, we compared models’ widely applicable information criterion (WAIC^100^). We then contrasted predictions generated from the posterior distribution of the best fit model, Model 1.3 (Supplementary Table 1), to determine the impact of harshness, variability, and reward value on participants’ propensity to switch jars. Specifically, we computed contrasts between the Stable Not Harsh—Stable Harsh conditions and the Variable Not Harsh—Variable Harsh conditions. Recall, for these contrasts, we broadly identified elective and responsive switching as switches that occurred immediately after finding high (55–100) and low (0–45) value rewards, respectively. This designation is somewhat arbitrary; however, as it is unclear where participants’ threshold for strategy “failure” might be, we chose rather conservative groupings bisecting the possible reward distribution (0–100).

### Study 2 Incentive switching analyses

Study 2 Switching Analyses were identical to Study 1, with the addition of the, between participants, monetary incentive variable. Thus, Model 6.0 and Model 6.1 were identical to Models 1.0 and 1.1. But Model 6.2 allowed the intercept and reward value slope to also depend on participants’ incentive [No added incentive (1) and Monetary incentive (0], with maximal random effects structure but no interaction between variability, harshness, or incentive. Model 6.3 allowed the intercept and reward value slope to depend also upon the interactions between variability and harshness, variability and incentive, and incentive and harshness. Model 6.4 then allowed the intercept and reward value slope to depend also upon the combined interaction between variability, harshness, and incentive. Based on our hypotheses and predictions, we expected Model 6.4 to best fit the data in Study 2. To determine the best fit model, we compared models’ widely applicable information criterion (WAIC^100^). We then contrasted predictions generated from the posterior distribution of the best fit model, Model 6.4 (Supplementary Table 1), to determine the impact of harshness, variability, incentive, and reward value on participants’ propensity to switch jars. Contrasts were computed both for the effect of harshness (e.g., Stable No Money Not Harsh—Stable No Money Harsh conditions, etc.) as well as for incentive (e.g., Stable Easy Money—Stable Easy No Money, etc.).

### Studies 1 and 2 Reinforcement learning model analyses

We ran a Bayesian multi-level reinforcement learning model as implemented by Deffner et al.^59^ and also used in^60,61^. The model assumed that participants choose among jars based on latent values (or “attractions”) they assign to them as a result of their prior experiences and update the values through a standard Rescorla-Wagner updating rule^101^. For each variability condition *k* and harshness condition *l*, we calculated participant-specific measures of learning *φ*_*k,l,j*_ and elective exploration* λ*_*k,l,j*_. Specifically, the learning parameter represented the speed at which participant *j* updated their preferences for each jar *i*, *A*_*i,j,t*_*,* based on their recent experiences with jar payoffs *π*_*i,j,t*_. Higher values of *φ*_*k,l,j*_ correspond to faster learning.

$$A_{i,j,t + 1} = (1 - \varphi_{k,l,j} )A_{i,j,t} + \varphi_{k,l,j} \pi_{i,j,t}$$

The *λ*_*k,l,j*_ parameter is a measure of the extent to which participants' jar choices were guided by their jar preferences. In other words, how strictly participants used the jar with the highest expected payoff versus exploring alternatives. Here, we will refer to *λ*_*k,l,j*_ as an *elective exploration* parameter; however, it is also referred to as *exploration rate* or *inverse temperature* in the reinforcement learning literature^99^. Lower values of *λ*_*k,l,j*_ correspond to increased elective exploration with individuals choosing at random when *λ*_*k,l,j*_ = 0.

$$P_{i, t + 1} = \frac{{exp\left( {\lambda_{k,l,j} A_{i,j,t} } \right)}}{{\mathop \sum \nolimits_{m = 1}^{2} exp\left( {\lambda_{k,l,j} A_{m,j,t} } \right)}}$$

Study 2 Reinforcement Learning Model Analyses were identical to Study 1, with the inclusion of the between-participants effect of monetary incentive. This means we now estimated learning *φ*_*k,l,m,j*_ and elective exploration* λ*_*k,l,m,j*_ now also separately for each incentive condition *m*.

### Supplementary Information

Supplementary Information.

## Data Availability

All data and analysis scripts are available in a Github repository at https://github.com/sarahpopecaldwell/HarshFlex_BanditJars.
