# Supplementary material for: Variability and harshness shape flexible strategy-use in support of the constrained flexibility framework
Source: Sci Rep. 2024 Mar 27;14:7236. doi: 10.1038/s41598-024-57800-w (PMC10973413; doi:10.1038/s41598-024-57800-w)
Supplement: Supplementary file 1 — Supplementary Information. [file 41598_2024_57800_MOESM1_ESM.pdf]

Supplementary Table 1: Models 1.0, 1.1, 1.2, and 1.3 investigating the impact of reward value, harshness, and variability on participants' likelihood of switching jars in Study 1. a) WAIC estimates and standard error comparison. b) Model estimates and 95% credible intervals. c) Contrasted predictions between Harsh and Not Harsh conditions for responsive and elective switching, characterized by switching after finding rewards between 0-45 and 55-100 units, respectively, from the best fit model. d) Contrasted predictions between Harsh and Not Harsh conditions for responsive switching, characterized by switching after finding rewards between 0-25, from the best fit model.

**a)**		*WAIC diff*	*se*
	Model 1.3	0	0
	Model 1.2	-93.2	14.4
	Model 1.1	-1446.4	51.1
	Model 1.0	-6864.6	95.1

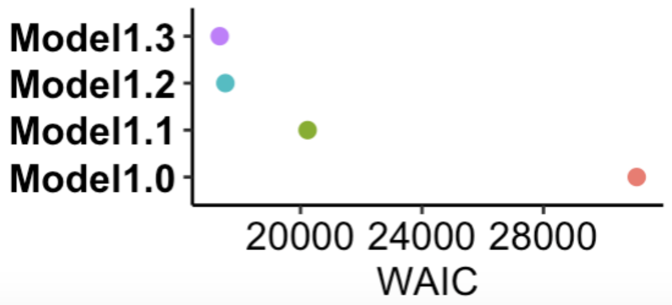

**b)**	Model 1.0		Model 1.1		Model 1.2		**Model 1.3**	
Predictors	*Log-Odds*	*CI (95%)*	*Log-Odds*	*CI (95%)*	*Log-Odds*	*CI (95%)*	*Log-Odds*	*CI (95%)*
Intercept	-1.12	-1.31 – -0.94	4.39	3.95 – 4.86	8.12	7.37 – 8.96	7.67	6.88 – 8.53
rewardfound			-0.08	-0.09 – -0.08	-0.14	-0.15 – -0.13	-0.13	-0.14 – -0.12
harshness: harsh					0.11	-0.33 – 0.54	0.78	0.10 – 1.46
universe: uv					-4.54	-5.32 – -3.81	-4.02	-4.83 – -3.23
harshnessharsh:rewardfound					-0.00	-0.01 – 0.00	-0.01	-0.02 – -0.00
universeuv:rewardfound					0.07	0.06 – 0.08	0.06	0.05 – 0.08
harshnessharsh:universeuv							-0.72	-1.31 – -0.14
harshnessharsh:universeuv:rewardfound							0.01	-0.00 – 0.02

**c)**	**Not Harsh – Harsh**	*HDP 2.5%*	*HDP 97.5%*	**Not Harsh – Harsh**	*HDP 2.5%*	*HDP 97.5%*
	Stable Responsive (0-45)	**-0.05**	**0.00**	Stable Elective (55-100)	-0.08	0.03
	Variable Responsive (0-45)	-0.03	0.07	Variable Elective (55-100)	-0.02	0.11

**d)**	**Not Harsh – Harsh**	*HDP 2.5%*	*HDP 97.5%*
	Stable Responsive (0-25)	**-0.01**	**0**
	Variable Responsive (0-25)	-0.02	0.03

Supplementary Table 2: Models 2.0, 2.1, 2.2, and 2.3 investigating the impact of reward value, harshness, and variability on participants' likelihood of switching jars including age, sex, and specific reward schedule in Study 1. a) WAIC estimates and standard error comparison. b) Model estimates and 95% credible intervals. c) Contrasted predictions between Harsh and Not Harsh conditions for responsive and elective switching, characterized by switching after finding rewards between 0-45 and 55-100 units, respectively, from the best fit model.

**a)**	*WAIC diff*	*se*
Model 2.3	0	0
Model 2.2	-93.8	14.5
Model 2.1	-1446.9	51.1
Model 2.0	-6864.6	192.52

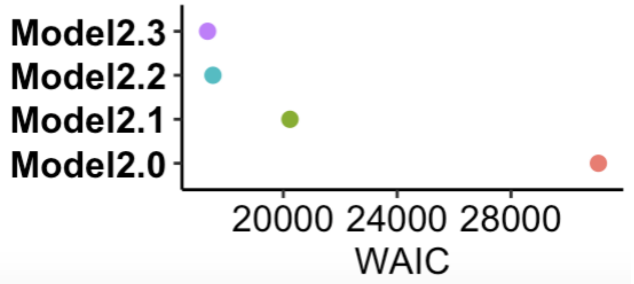

**b)**	Model 2.0		Model 2.1		Model 2.2		**Model 2.3**	
	Log-Odds	*CI (95%)*	*Log-Odds*	*CI (95%)*	*Log-Odds*	*CI (95%)*	*Log-Odds*	*CI (95%)*
Intercept	-1.12	-1.29 – -0.95	4.37	3.75 – 5.01	8.17	7.31 – 9.08	7.74	6.89 – 8.64
rewardfound			-0.08	-0.09 – -0.08	-0.14	-0.15 – -0.13	-0.13	-0.14 – -0.12
age			-0.03	-0.25 – 0.18	0.02	-0.18 – 0.23	0.03	-0.18 – 0.23
sex			0.10	-0.36 – 0.54	0.35	-0.08 – 0.79	0.39	-0.04 – 0.81
looplabel: loop2			0.03	-0.74 – 0.76	0.11	-0.61 – 0.82	0.17	-0.53 – 0.89
looplabel: loop3			0.01	-0.68 – 0.71	-0.35	-1.01 – 0.34	-0.44	-1.12 – 0.23
looplabel: loop4			-0.35	-1.08 – 0.33	-0.39	-1.07 – 0.28	-0.38	-1.07 – 0.29
looplabel: loop5			0.28	-0.37 – 0.94	0.32	-0.32 – 0.95	0.34	-0.30 – 0.99
harshness: harsh					0.12	-0.33 – 0.56	0.79	0.12 – 1.46
universe: uv					-4.54	-5.32 – -3.79	-4.03	-4.83 – -3.27
harshnessharsh:rewardfound					-0.00	-0.01 – 0.00	-0.01	-0.02 – -0.00
universeuv:rewardfound					0.07	0.06 – 0.08	0.06	0.05 – 0.08
harshnessharsh:universeuv							-0.72	-1.31 – -0.14
harshnessharsh:universeuv:rewardfound							0.01	-0.00 – 0.02

**c)**	**Not Harsh – Harsh**	*HDP 2.5%*	*HDP 97.5%*	**Not Harsh – Harsh**	*HDP 2.5%*	*HDP 97.5%*
	Stable Responsive (0-45)	**-0.06**	**0.00**	Stable Elective (55-100)	-0.08	0.02
	Variable Responsive (0-45)	-0.04	0.07	Variable Elective (55-100)	-0.01	0.10

Supplementary Table 3: Models 3.0, 3.1, 3.2, and 3.3 investigating the impact of reward value, harshness, and variability on participants' likelihood of switching jars for participants who reported that the Harsh condition was more stressful than the Not Harsh condition in Study 1. a) WAIC estimates and standard error comparison. b) Model estimates and 95% credible intervals. c) Contrasted predictions between Harsh and Not Harsh conditions for responsive and elective switching, characterized by switching after finding rewards between 0-45 and 55-100 units, respectively, from the best fit model.

**a)**		*WAIC diff*	*se*
	Model 3.3	0	0
	Model 3.2	-51.5	11.3
	Model 3.1	-853.1	39.5
	Model 3.0	-4568.4	159.82

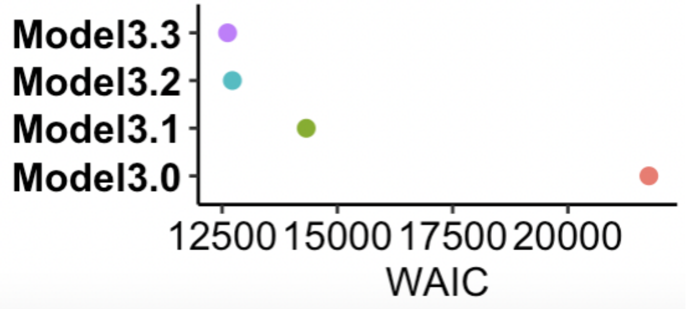

**b)**	Model 3.0		Model 3.1		Model 3.2		**Model 3.3**	
	Log-Odds	*CI (95%)*	*Log-Odds*	*CI (95%)*	*Log-Odds*	*CI (95%)*	*Log-Odds*	*CI (95%)*
Intercept	-1.14	-1.31 – -0.95	4.34	3.85 – 4.83	7.70	6.88 – 8.62	7.24	6.40 – 8.12
rewardfound			-0.08	-0.09 – -0.08	-0.13	-0.14 – -0.12	-0.12	-0.14 – -0.11
harshness: harsh					0.02	-0.39 – 0.44	0.76	0.07 – 1.46
universe: uv					-4.06	-4.91 – -3.25	-3.57	-4.43 – -2.74
harshnessharsh:rewardfound					-0.01	-0.01 – 0.00	-0.01	-0.02 – -0.00
universeuv:rewardfound					0.06	0.05 – 0.07	0.06	0.04 – 0.07
harshnessharsh:universeuv							-0.66	-1.27 – -0.07
harshnessharsh:universeuv:rewardfound							0.00	-0.01 – 0.02

**c)**	**Not Harsh – Harsh**	*HDP 2.5%*	*HDP 97.5%*	**Not Harsh – Harsh**	*HDP 2.5%*	*HDP 97.5%*
	Stable Responsive (0-45)	-0.04	0	Stable Elective (55-100)	-0.04	0.06
	Variable Responsive (0-45)	-0.02	0.09	Variable Elective (55-100)	**0.00**	**0.15**

Supplementary Table 4: Models 4.0, 4.1, 4.2, and 4.3 investigating the impact of reward value, harshness, and variability on participants' likelihood of switching jars for the first round of data collection with heavy sampling bias (see addendum to the preregistration: Pope, 2021b). a) WAIC estimates and standard error comparison. b) Model estimates and 95% credible intervals. c) Contrasted predictions between Harsh and Not Harsh conditions for responsive and elective switching, characterized by switching after finding rewards between 0-45 and 55-100 units, respectively, from the best fit model.

**a)**	*WAIC diff*	*se*
Model 4.3	0	0
Model 4.2	-96.6	14.3
Model 4.1	-1123.2	46.8
Model 4.0	-6148.1	95.1

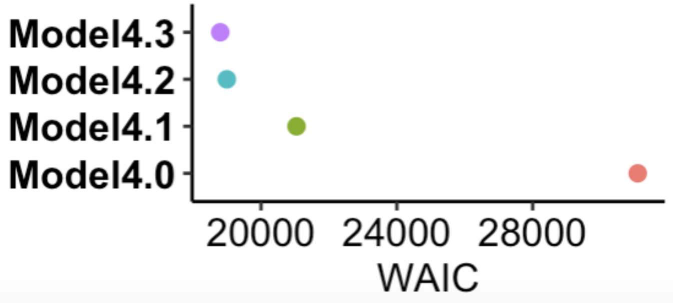

**b)**	Model 4.0		Model 4.1		Model 4.2		**Model 4.3**	
	Log-Odds	*CI (95%)*	*Log-Odds*	*CI (95%)*	*Log-Odds*	*CI (95%)*	*Log-Odds*	*CI (95%)*
Intercept	-1.14	-1.26 – -1.00	4.11	3.75 – 4.47	7.30	6.63 – 8.03	6.98	6.28 – 7.72
rewardfound			-0.08	-0.09 – -0.08	-0.12	-0.13 – -0.11	-0.12	-0.13 – -0.11
harshness: harsh					0.05	-0.29 – 0.39	0.37	-0.17 – 0.92
universe: uv					-3.65	-4.33 – -3.01	-3.19	-3.91 – -2.52
harshnessharsh:rewardfound					-0.00	-0.01 – 0.00	-0.01	-0.01 – 0.00
universeuv:rewardfound					0.05	0.04 – 0.06	0.04	0.03 – 0.05
harshnessharsh:universeuv							-0.51	-1.04 – 0.02
harshnessharsh:universeuv:rewardfound							0.01	-0.00 – 0.02

**c)**	*HDP 2.5%*	*HDP 97.5%*	**Not Harsh – Harsh**	*HDP 2.5%*	*HDP 97.5%*
**Not Harsh – Harsh**					
Stable Responsive (0-45)	-0.03	0.01	Stable Elective (55-100)	-0.03	0.05
Variable Responsive (0-45)	-0.02	0.05	Variable Elective (55-100)	-0.04	0.05

Supplementary Table 5: Models 5.0, 5.1, 5.2, and 5.3 investigating the impact of reward value, harshness, and variability on participants' likelihood of switching jars in Study 2. a) WAIC estimates and standard error comparison. b) Model estimates and 95% credible intervals. c) Contrasted predictions between Harsh and Not Harsh conditions for responsive and elective switching, characterized by switching after finding rewards between 0-45 and 55-100 units, respectively, from the best fit model. d) Contrasted predictions between Harsh and Not Harsh conditions for responsive switching, characterized by switching after finding rewards between 0-25, from the best fit model.

a)		*WAIC diff*	*se*
	Model 5.3	0	0
	Model 5.2	-161.1	18.7
	Model 5.1	-2561.6	68.5
	Model 5.0	-12507.9	134.2

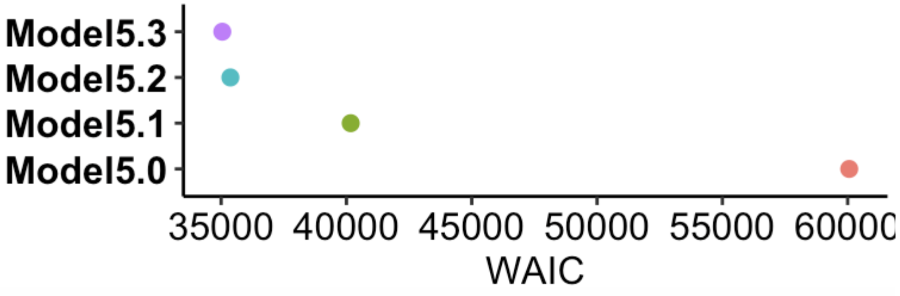

b)	Model 5.0		Model 5.1		Model 5.2		**Model 5.3**	
	Log-Odds	*CI (95%)*	*Log-Odds*	*CI (95%)*	*Log-Odds*	*CI (95%)*	*Log-Odds*	*CI (95%)*
Intercept	-1.24	-1.36 – -1.13	4.08	3.79 – 4.37	8.99	8.37 – 9.66	8.69	8.04 – 9.39
rewardfound			-0.08	-0.09 – -0.08	-0.15	-0.16 – -0.14	-0.14	-0.15 – -0.13
harshness: harsh					0.08	-0.25 – 0.40	0.26	-0.26 – 0.79
universe: uv					-5.28	-5.89 – -4.70	-4.93	-5.57 – -4.28
harshnessharsh:rewardfound					-0.00	-0.01 – 0.00	-0.01	-0.01 – 0.00
universeuv:rewardfound					0.07	0.06 – 0.08	0.06	0.05 – 0.07
harshnessharsh:universeuv							-0.20	-0.68 – 0.31
harshnessharsh:universeuv:rewardfound							0.00	-0.01 – 0.01

c)	**Not Harsh – Harsh**	*HDP 2.5%*	*HDP 97.5%*	**Not Harsh – Harsh**	*HDP 2.5%*	*HDP 97.5%*
	Stable Responsive (0-45)	-0.01	0.01	Stable Elective (55-100)	-0.02	0.06
	Variable Responsive (0-45)	-0.02	0.05	Variable Elective (55-100)	**-0.00**	**0.06**

d)	**Not Harsh – Harsh**	*HDP 5%*	*HDP 95%*
	Stable Responsive (0-25)	0.00	0.00
	Variable Responsive (0-25)	-0.02	0.02

Supplementary Table 6: Models 6.0, 6.1, 6.2, 6.3 & 6.4 i investigating the impact of reward value, incentive, harshness, and variability, on participants' likelihood of switching jars between Study 1 and Study 2. a) WAIC estimates and standard error comparison. b) Model estimates and 95% credible intervals. c) Contrasted predictions between Harsh and Not Harsh conditions for responsive and elective switching, characterized by switching after finding rewards between 0-45 and 55-100 units, respectively, from the best fit model. Note, data from both Study 1 and Study 2 are included.

a)

	WAIC diff	*se*
Model 6.4	0	0
Model 6.3	-3.3	0.8
Model 6.2	-257.2	23.7
Model 6.1	-4045.8	86.5
Model 6.0	-19411.4	165.0

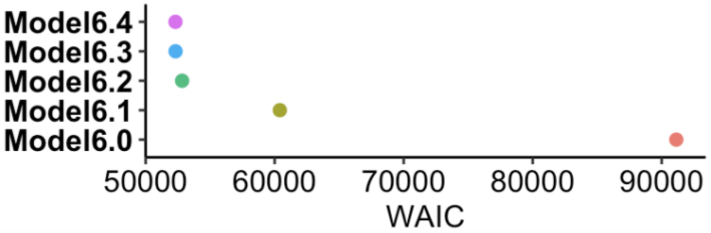

b)

	Model 6.0		Model 6.1		Model 6.2		Model 6.3		**Model 6.4**	
	Log-Odds	*CI (95%)*	*Log-Odds*	*CI (95%)*	*Log-Odds*	*CI (95%)*	*Log-Odds*	*CI (95%)*	*Log-Odds*	*CI (95%)*
Intercept	-1.20	-1.30 – -1.11	4.19	3.93 – 4.45	9.35	8.76 – 9.98	8.99	8.34 – 9.65	8.99	8.36 – 9.66
Rewardfound			-0.08	-0.09 – -0.08	-0.15	-0.16 – -0.14	-0.15	-0.16 – -0.14	-0.15	-0.16 – -0.14
harshness: harsh					0.06	-0.22 – 0.34	0.48	-0.03 – 0.99	0.47	-0.05 – 0.98
universe: uv					-5.68	-6.25 – -5.16	-5.15	-5.77 – -4.54	-5.15	-5.77 – -4.55
incentive: nomoney					0.04	-0.49 – 0.55	-0.08	-0.84 – 0.71	-0.08	-0.86 – 0.69
rewardfound:harshnessharsh					-0.00	-0.01 – 0.00	-0.01	-0.01 – -0.00	-0.01	-0.01 – -0.00
rewardfound:universeuv					0.08	0.07 – 0.09	0.07	0.06 – 0.08	0.07	0.06 – 0.08
rewardfound:incentivenomoney					0.00	-0.01 – 0.01	-0.00	-0.01 – 0.01	-0.00	-0.01 – 0.01
universeuv:harshnessharsh							-0.51	-0.96 – -0.06	-0.51	-0.96 – -0.06
universeuv:incentivenomoney							-0.14	-0.73 – 0.46	-0.15	-0.75 – 0.44
harshnessharsh:incentivenomoney							0.09	-0.36 – 0.54	0.12	-0.34 – 0.56
rewardfound:universeuv:harshnessharsh							0.01	-0.00 – 0.01	0.01	-0.00 – 0.01
rewardfound:universeuv:incentivenomoney							0.01	0.00 – 0.02	0.01	0.00 – 0.03
rewardfound:harshnessharsh:incentivenomoney							-0.00	-0.01 – 0.01	-0.00	-0.01 – 0.01
universeuv:harshnessharsh:incentivenomoney							-0.02	-0.11 – 0.08	-0.01	-0.11 – 0.09
rewardfound:universeuv:harshnessharsh:incentivenomoney									-0.00	-0.01 – 0.01

c)

**No – Monetary Incentive**	*HDP 2.5%*	*HDP 97.5%*	**No – Monetary Incentive**	*HDP 2.5%*	*HDP 97.5%*
Stable Responsive (0-45) Not Harsh	-0.02	0.01	Stable Responsive (0-45) Harsh	-0.01	0.01
Stable Elective (55-100) Not Harsh	-0.10	0.03	Stable Elective (55-100) Harsh	-0.09	0.03
Variable Responsive (0-45) Not Harsh	-0.03	0.12	Variable Responsive (0-45) Harsh	-0.03	0.11
Variable Elective (55-100) Not Harsh	**0.01**	**0.19**	Variable Elective (55-100) Harsh	**0.00**	**0.14**

Supplementary Table 7: Models 7.0, 7.1, 7.2, 7.3 & 7.4 investigating the impact of reward value, incentive, harshness, and variability, on participants' likelihood of switching jars including age, sex, and specific reward schedule, between Study 1 and Study 2. a) WAIC estimates and standard error comparison. b) Model estimates and 95% credible intervals. c) Contrasted predictions between Harsh and Not Harsh conditions for responsive and elective switching, characterized by switching after finding rewards between 0-45 and 55-100 units, respectively, from the best fit model. Note, data from both Study 1 and Study 2 are included.

a)

	WAIC diff	*se*
Model 7.3	0	0
Model 7.4	-2.9	0.7
Model 7.2	-256.3	23.7
Model 7.1	-4044.6	86.6
Model 7.0	-19411.2	165.1

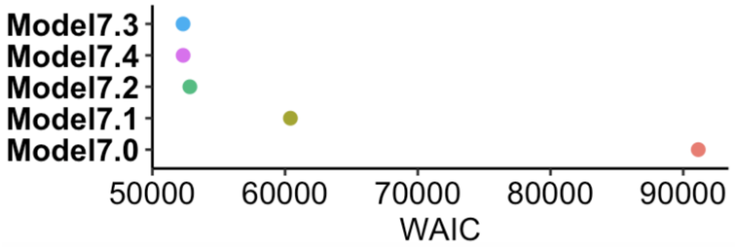

b)

	Model 7.0		Model 7.1		Model 7.2		Model 7.3		**Model 7.4**	
	Log-Odds	*CI (95%)*	*Log-Odds*	*CI (95%)*	*Log-Odds*	*CI (95%)*	*Log-Odds*	*CI (95%)*	*Log-Odds*	*CI (95%)*
Intercept	-1.20	-1.30 – -1.11	4.22	3.86 – 4.58	9.44	8.81 – 10.09	9.10	8.41 – 9.80	9.09	8.41 – 9.79
rewardfound			-0.08	-0.09 – -0.08	-0.15	-0.16 – -0.14	-0.15	-0.16 – -0.14	-0.15	-0.16 – -0.14
age			-0.10	-0.23 – 0.02	-0.10	-0.23 – 0.04	-0.09	-0.23 – 0.04	-0.10	-0.23 – 0.04
sex			0.13	-0.14 – 0.39	0.17	-0.08 – 0.42	0.17	-0.08 – 0.42	0.18	-0.07 – 0.42
looplabel: loop2			-0.11	-0.53 – 0.31	-0.14	-0.54 – 0.28	-0.13	-0.55 – 0.27	-0.14	-0.53 – 0.27
looplabel: loop3			0.08	-0.33 – 0.50	-0.12	-0.51 – 0.28	-0.17	-0.57 – 0.21	-0.17	-0.56 – 0.22
looplabel: loop4			-0.23	-0.63 – 0.20	-0.10	-0.47 – 0.30	-0.07	-0.46 – 0.32	-0.07	-0.47 – 0.33
looplabel: loop5			0.06	-0.33 – 0.46	0.03	-0.35 – 0.42	0.01	-0.37 – 0.40	0.02	-0.36 – 0.39
harshness: harsh					0.06	-0.23 – 0.33	0.49	-0.02 – 0.99	0.48	-0.04 – 0.99
universe: uv					-5.67	-6.24 – -5.14	-5.16	-5.80 – -4.55	-5.16	-5.76 – -4.55
incentive: nomoney					-0.05	-0.59 – 0.47	-0.15	-0.91 – 0.64	-0.14	-0.89 – 0.61
rewardfound:harshnessharsh					-0.00	-0.01 – 0.00	-0.01	-0.01 – -0.00	-0.01	-0.01 – -0.00
rewardfound:universeuv					0.08	0.07 – 0.09	0.07	0.06 – 0.08	0.07	0.06 – 0.08
rewardfound:incentivenomoney					0.00	-0.01 – 0.01	-0.00	-0.01 – 0.01	-0.00	-0.01 – 0.01
universeuv:harshnessharsh							-0.51	-0.97 – -0.06	-0.51	-0.96 – -0.05
universeuv:incentivenomoney							-0.13	-0.74 – 0.43	-0.15	-0.73 – 0.44
harshnessharsh:incentivenomoney							0.09	-0.35 – 0.54	0.11	-0.34 – 0.57
rewardfound:universeuv:harshnessharsh							0.01	-0.00 – 0.01	0.01	-0.00 – 0.01
rewardfound:universeuv:incentivenomoney							0.01	0.00 – 0.02	0.01	0.00 – 0.03
rewardfound:harshnessharsh:incentivenomoney							-0.00	-0.01 – 0.01	-0.00	-0.01 – 0.01

Supplementary Table 7 continued:

universeuv:harshnessharsh:incentivenomoney	-0.02	-0.11 – 0.08	-0.01	-0.11 – 0.09
rewardfound:universeuv:harshnessharsh:incentivenomoney			-0.00	-0.01 – 0.01

c)	**No – Monetary Incentive**	*HDP 2.5%*	*HDP 97.5%*	**No – Monetary Incentive**	*HDP 2.5%*	*HDP 97.5%*
	Stable Responsive (0-45) Not Harsh	-0.03	0.00	Stable Responsive (0-45) Harsh	-0.02	0.00
	Stable Elective (55-100) Not Harsh	-0.11	0.01	Stable Elective (55-100) Harsh	-0.11	0.01
	Variable Responsive (0-45) Not Harsh	-0.05	0.09	Variable Responsive (0-45) Harsh	-0.04	0.09
	Variable Elective (55-100) Not Harsh	**0.00**	**0.15**	Variable Elective (55-100) Harsh	**0.00**	**0.13**

Supplementary Table 8: Models 8.0, 8.1, 8.2, 8.3 & 8.4 investigating the impact of reward value, incentive, harshness, and variability, on participants' likelihood of switching jars for participants who reported that the Harsh condition was more stressful than the Not Harsh condition, between Study 1 and Study 2. a) WAIC estimates and standard error comparison. b) Model estimates and 95% credible intervals. c) Contrasted predictions between Harsh and Not Harsh conditions for responsive and elective switching, characterized by switching after finding rewards between 0-45 and 55-100 units, respectively, from the best fit model. Note, data from both Study 1 and Study 2 are included.

a)

	WAIC diff	*se*
Model 8.3	0	0
Model 8.4	-0.4	0.8
Model 8.2	-177.7	19.7
Model 8.1	-2589.2	69.
Model 8.0	-13035.3	136.6

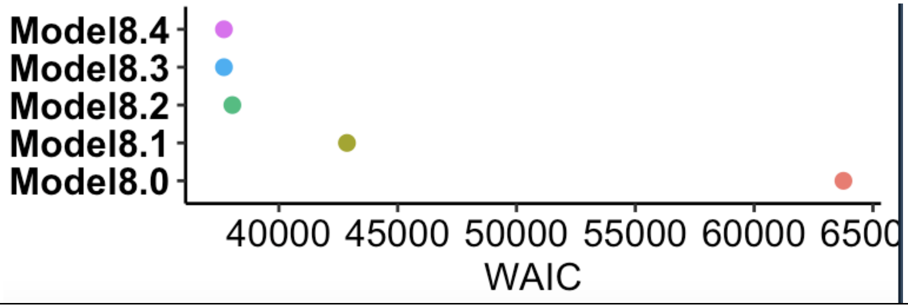

b)

	Model 8.0		Model 8.1		Model 8.2		Model 8.3		**Model 8.4**	
	Log-Odds	*CI (95%)*	*Log-Odds*	*CI (95%)*	*Log-Odds*	*CI (95%)*	*Log-Odds*	*CI (95%)*	*Log-Odds*	*CI (95%)*
Intercept	-1.20	-1.30 – -1.10	4.06	3.78 – 4.34	8.93	8.26 – 9.64	8.61	7.91 – 9.34	8.61	7.93 – 9.37
rewardfound			-0.08	-0.09 – -0.08	-0.15	-0.16 – -0.14	-0.14	-0.15 – -0.13	-0.14	-0.15 – -0.13
harshness: harsh					0.10	-0.18 – 0.37	0.53	-0.02 – 1.07	0.50	-0.05 – 1.04
universe: uv					-5.49	-6.13 – -4.90	-4.99	-5.68 – -4.34	-4.98	-5.67 – -4.33
incentive: nomoney					0.21	-0.36 – 0.78	0.16	-0.67 – 0.97	0.12	-0.72 – 0.96
rewardfound:harshnessharsh					-0.01	-0.01 – -0.00	-0.01	-0.02 – -0.00	-0.01	-0.02 – -0.00
rewardfound:universeuv					0.08	0.07 – 0.08	0.07	0.06 – 0.08	0.06	0.05 – 0.08
rewardfound:incentivenomoney					-0.00	-0.01 – 0.01	-0.00	-0.01 – 0.01	-0.00	-0.02 – 0.01
universeuv:harshnessharsh							-0.59	-1.08 – -0.07	-0.59	-1.08 – -0.10
universeuv:incentivenomoney							-0.06	-0.66 – 0.54	-0.08	-0.69 – 0.55
harshnessharsh:incentivenomoney							0.01	-0.44 – 0.45	0.10	-0.36 – 0.57
rewardfound:universeuv:harshnessharsh							0.01	-0.00 – 0.02	0.01	0.00 – 0.02
rewardfound:universeuv:incentivenomoney							0.01	-0.00 – 0.02	0.01	-0.00 – 0.02
rewardfound:harshnessharsh:incentivenomoney							-0.00	-0.01 – 0.01	-0.00	-0.01 – 0.01
universeuv:harshnessharsh:incentivenomoney							-0.02	-0.12 – 0.08	-0.01	-0.11 – 0.09
rewardfound:universeuv:harshnessharsh:incentivenomoney									-0.01	-0.01 – 0.00

c)

**No – Monetary Incentive**	*HDP 2.5%*	*HDP 97.5%*	**No – Monetary Incentive**	*HDP 2.5%*	*HDP 97.5%*
Stable Responsive (0-45) Not Harsh	-0.02	0.01	Stable Responsive (0-45) Harsh	-0.01	0.02
Stable Elective (55-100) Not Harsh	-0.09	0.05	Stable Elective (55-100) Harsh	-0.06	0.07
Variable Responsive (0-45) Not Harsh	-0.03	0.13	Variable Responsive (0-45) Harsh	-0.03	0.10
Variable Elective (55-100) Not Harsh	**0.00**	**0.18**	Variable Elective (55-100) Harsh	-0.02	0.10

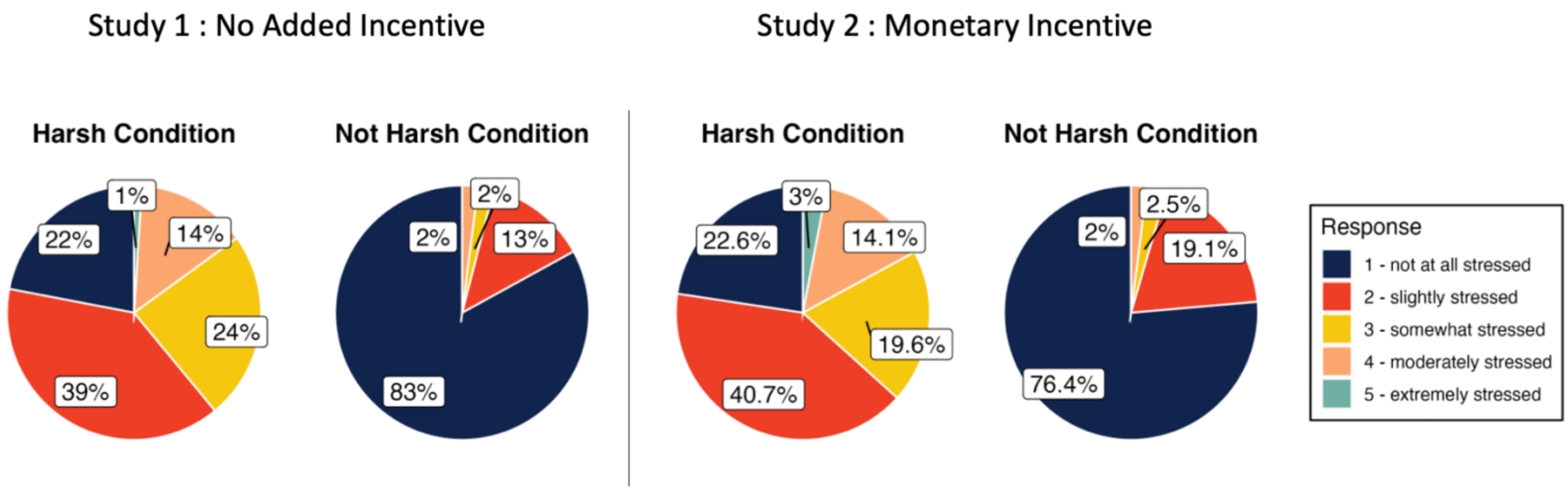

Supplementary Figure 1. Participants' self-reported feelings of stress during the Harsh and Not Harsh Conditions in Study 1 and Study 2.

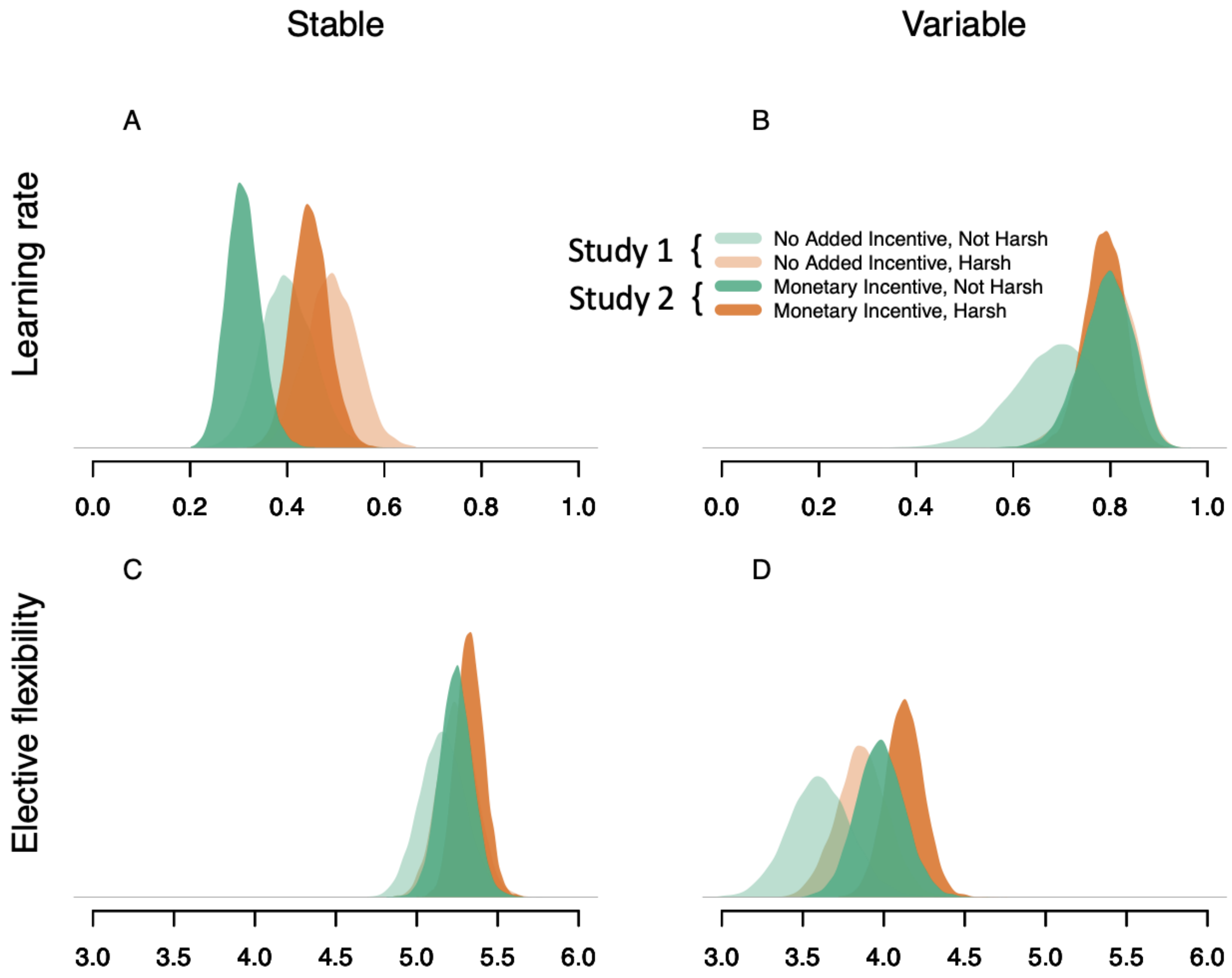

Supplementary Figure 2. Studies 1 and 2 Reinforcement Learning Model results <u>for participants' who reported that the Harsh condition was more stressful than the Not Harsh condition</u>. Posterior probability distributions in Stable and Variable reward environments for participants' learning rate (A-B) and elective exploration parameters (C-D) in Study 1: No Added Incentive - Not Harsh, No Added Incentive – Harsh and Study 2: Monetary Incentive - Not Harsh and Monetary Incentive - Harsh conditions.
